# A predicted protein, KIAA0247, is a cell cycle modulator in colorectal cancer cells under 5-FU treatment

**DOI:** 10.1186/1479-5876-9-82

**Published:** 2011-05-28

**Authors:** Chi-Jung Huang, Shung-Haur Yang, Shih-Ming Huang, Chih-Ming Lin, Chih-Cheng Chien, Yan-Chu Chen, Chia-Long Lee, Hao-Han Wu, Chun-Chao Chang

**Affiliations:** 1 School of Medicine, Fu Jen Catholic University, New Taipei 24205, Taiwan; 2 Department of Biochemistry, National Defense Medical Center, Taipei 11490, Taiwan; 3 Department of Medical Research, Cathay General Hospital, Taipei 10630, Taiwan; 4 Department of Surgery, Taipei-Veterans General Hospital and School of Medicine, National Yang Ming University, Taipei 11217, Taiwan; 5 Department of Surgery, Cathay General Hospital, Taipei 10630, Taiwan; 6 Department of Anesthesiology, Sijhih Cathay General Hospital, New Taipei 22174, Taiwan; 7 Department of Internal Medicine, Hsinchu Cathay General Hospital, Hsinchu 30060, Taiwan; 8 Division of Gastroenterology and Hepatology, Department of Internal Medicine, Taipei Medical University Hospital and Department of Internal Medicine, School of Medicine, College of Medicine, Taipei Medical University, Taipei 11031, Taiwan

## Abstract

**Background:**

Colorectal cancer (CRC) is the predominant gastrointestinal malignancy and the leading cause of cancer death. The identification of genes related to CRC is important for the development of successful therapies and earlier diagnosis.

**Methods:**

Molecular analysis of feces was evaluated as a potential method for CRC detection. Expression of a predicted protein with unknown function, KIAA0247, was found in feces evaluated using specific quantitative real-time polymerase chain reaction. Its cellular function was then analyzed using immunofluorescent staining and the changes in the cell cycle in response to 5-fluorouracil (5-FU) were assessed.

**Results:**

Gastrointestinal tissues and peripheral blood lymphocytes ubiquitously expressed KIAA0247. 56 CRC patients fell into two group categories according to fecal KIAA0247 mRNA expression levels. The group with higher fecal KIAA0247 (*n *= 22; ≥ 0.4897) had a significantly greater five-year overall survival rate than the group with lower fecal KIAA0247 (*n *= 30; < 0.4897) (66.0 ± 11.6%; *p *= 0.035, log-rank test). Fecal expression of KIAA0247 inversely related to CRC tumor size (Kendall's tau-b = -0.202; *p *= 0.047). Immunofluorescent staining revealed that the cytoplasm of CRC cells evenly expresses KIAA0247 without 5-FU treatment, and KIAA0247 accumulates in the nucleus after 40 μM 5-FU treatment. In HCT116 p53^-/- ^cells, which lack p53 cell cycle control, the proportion of cells in the G2/M phase was larger (13%) in KIAA0247-silent cells than in the respective shLuc control (10%) and KIAA0247-overexpressing cells (7%) after the addition of low dose (40 μM) 5-FU. Expression of three cyclin genes (cyclin A2, cyclin B1, and cyclin B2) also downregulated in the cells overexpressing KIAA0247.

**Conclusions:**

This is the first description of a linkage between KIAA0247 and CRC. The study's data demonstrate overexpression of KIAA0247 associates with 5-FU therapeutic benefits, and also identify the clinical significance of fecal KIAA0247 in CRC.

## Background

Colorectal cancer (CRC) is the predominant gastrointestinal malignancy and the leading cause of cancer death [1]. CRC usually arises as a consequence of the accumulation of genetic and epigenetic alterations in colonic epithelial cells during neoplastic transformation [2]. The identification of CRC-related genes is important for the development of successful therapies and earlier diagnosis [3-5].

Genes involved in cell growth, cell cycle, apoptosis, angiogenesis, or invasion could have a crucial role in CRC tumorigenesis [6,7]. In particular, some promising targets responsible for the control of cell cycle progression have attracted a great deal of attention for drug discovery [8,9]. In recent decades, researchers developed several agents with the function of regulating the degree of cell cycle arrest for cancer treatment [10,11]. Enhancement of the effects of defects in the G2/M arrest checkpoint that make a damaged cell enter mitosis and undergo apoptosis might increase the effective cytotoxicity of chemotherapy [8].

The novel gene, KIAA0247, previously identified as one of the CRC-related candidates, is a speculated target of the tumor suppressor gene, p53, because of a p53-responsive element in the promoter region [12,13]. This implies that KIAA0247 might participate in the p53 pathway of CRC tumorigenesis. Previous studies have identified that many molecules have altered expression in the feces of CRC patients [14,15]; some of these novel candidate genes with unknown function. The detailed characteristics of KIAA0247 are still unknown. Further understanding of the cellular functions in CRC of this predicted protein may provide an alternative target for CRC treatment.

The present study, therefore, aimed to investigate the molecular function of KIAA0247 in CRC tumorigenesis. Firstly, the clinical significance of KIAA0247 was evaluated from fecal samples of CRC patients using specific quantitative real-time polymerase chain reaction (qRT-PCR). Its cellular function was then evaluated using immunofluorescent staining and the changes in the cell cycle in response to 5-fluorouracil (5-FU) were assessed. Results demonstrated that, in CRC patients, the expression of KIAA0247 influences the effects of treatment with 5-FU at a relatively low concentration.

## Methods

### Patients

Solid fecal samples (approximately 0.5 g) from 56 CRC patients from the Cathay General Hospital (CGH) or Taipei Veterans General Hospital were taken before surgery or any application of chemotherapy with Institutional Review Board (IRB)-approved informed consent at the CGH IRB. Follow-up data were obtained prospectively, and the mean follow-up time was 34.9 months (SD, 26.8; median, 23). The patients' initial tumor stage and other clinical information are listed in Table 1. Presence of distant metastasis was routinely confirmed by abdominal computed tomography.

**Table 1:** Analyses of mRNA levels of fecal KIAA0247 in clinical features

**Features**^a^		***n***^b^	Cases with higher mRNA levels of fecal KIAA0247 (>0.4897)	***p*-value**^c^
Age (years)				
	≤ 66.0	27	11 (40.7%)	0.874
	>66.0	28	12 (42.9%)	
Gender				
	Male	37	14 (37.8%)	0.290
	Female	19	10 (52.6%)	
Dukes' stages				
	A+B	28	14 (50.0%)	0.210
	C+D	27	9 (33.3%)	
Depth of invasion				
	T1+T2	24	14 (58.3%)	0.053
	T3+T4	31	10 (32.3%)	
Lymphatic invasion				
	N0	32	15 (46.9%)	0.568
	N1+N2+N3	23	9 (39.1%)	
Distant metastasis				
	No	34	18 (52.9%)	0.058
	Yes	22	6 (27.3%)	
Tumor location				
	Right	20	6 (30.0%)	
	Left	13	6 (46.2%)	0.341
	Rectum	19	10 (52.6%)	
CEA (ng/ml)				
	≤ 5	28	15 (53.6%)	0.072
	>5	27	8 (29.6%)	
CA19-9 (U/ml)				
	<37	34	15 (44.1%)	0.371
	≥ 37	19	6 (31.6%)	
Differentiation				
	Well/moderate	47	22 (46.8%)	0.065
	Poor	5	0 (0%)	

^a ^Median age, 66 years; age range, 40.3-89.5 years; CEA, carcinoembryonic antign; CA19-9, carbohydrate antigen 19-9.

^b ^Numbers of assessed cases are dependent on the available cases.

### Colonic cell lines and human multiple tissue cDNA

The p53-null HCT116 cell line (HCT116 p53^-/-^, a gift from Prof. Bert Vogelstein) was cultured in Dulbecco's modified Eagles medium with 5 mM glutamine according to routine culture procedures. The cDNAs of multiple gastrointestinal tissues and PBL for qRT-PCR were selected from the human multiple tissue cDNA panels (BD Biosciences Clontech, Mountain View, CA).

### Total RNA extraction and reverse transcription reaction

Total RNA from these cultured cells was extracted using the Easy Pure Total RNA Mini Kit (Bioman, Taipei, Taiwan) according to the manufacturer's instructions and fecal RNA was prepared as reportedly previously [16]. One microgram of cellular total RNA or fecal RNA was reverse transcribed to single-stranded cDNA using an oligo(dT)_12 _primer with the ABI Reverse Transcriptase Kit (Applied Biosystems, Carlsbad, CA) according to the manufacturer's protocol. Synthesized cDNA could be used directly in the following qRT-PCR analyses.

### qRT-PCR

The qRT-PCR for quantifying targets in multiple tissue cDNA, cellular cDNA, and fecal cDNA was performed using a TaqMan probe, from the Human Universal Probe Library (Roche Diagnostics, Mannheim, Germany), as described previously [17,18] except for fecal KIAA0247 (NM014734). To quantify fecal KIAA0247, the amount of each primer was elevated to 4 pmol in a 10 μL reaction volume. Each fecal sample run also included human reference cDNA (Clontech, Mountain View, CA) as a standard to estimate the relative expression levels in feces. The relative levels of expression of genes in various samples were determined by normalizing their expression to that of 18S ribosomal (r)RNA (X03205) [19]. The primers and universal probes used to quantify KIAA0247, cyclin A2 (NM001237), cyclin B1 (NM031966), and cyclin B2 (NM004701) are listed in Table 2.

**Table 2:** Primers and TaqMan probes for qRT-PCR

Gene name	Reference	**Primer sequence (5' to 3')**^a^	**Probe number**^b^
KIAA0247	NM014734	F: CTGCAGATTCAGAGAACAGTGAC	82
		R: CTCATGCTTCTTTCAACAGTGG	
			
Cyclin A2	NM001237	F: CCATACCTCAAGTATTTGCCATC	67
(CCNA2)		R: TCCAGTCTTTCGTATTAATGATTCAG	
			
Cyclin B1	NM031966	F: CATGGTGCACTTTCCTCCTT	18
(CCNB1)		R: AGGTAATGTTGTAGAGTTGGTGTCC	
			
Cyclin B2	NM004701	F: GCATTATCATCCTTCTAAGGTAGCA	4
(CCNB2)		R: TGTAATACTGCTGCTTTAAGTTCCA	

^a ^F, forward primer; R, reverse primer.

^b ^Probe number, from the Human Universal Probe Library of Roche Diagnostics, Mannheim, Germany.

### Lentivirus-mediated RNA interference (RNAi) and overexpression of KIAA0247

The lentiviral constructs encoding the siKIAA0247 hairpin (pLKO.1-KIAA0247: TRCN0000134410) for gene silencing (shKIAA0247) or the KIAA0247 cDNA for gene overexpression (overKIAA0247) were obtained from the National RNAi Core Facility located at the Institute of Molecular Biology/Genomic Research Center, Academia Sinica, Taiwan. pLKO.1-Luc (TRCN0000072246) acted as a control (shLuc) for the previously mentioned two lentiviruses. Infection of each lentivirus into colonic cells was performed as described previously. Changes in the expression of KIAA0247 were determined using qRT-PCR.

### Cell cycle analysis by flow cytometry

To determine the cellular effects of KIAA0247 in colonic cells, cell cycle analysis was performed using flow cytometry by analyzing the DNA content [20] of propidium iodide (PI)-stained nuclei as described previously [21]. Colonic cells transfected with shKIAA0247, shLuc, or overKIAA0247 were plated, at a density of 5 × 10^6 ^cells/well in 6-well dishes, and cultured for 24 h. These subconfluent cells were incubated with DNA analogue 5-FU (40 μM) (Sigma-Aldrich, St. Louis, MO) for another 24 h. The control cells were treated with medium alone. Thereafter, cells were trypsinized, washed twice with PBS, and fixed in 70% ethanol for 5 h at 4°C. These fixed cells were washed twice more with PBS, incubated with 1 μg/ml RNase A for 1 h at 37°C, and stained with 5 μg/ml PI for 1 h at room temperature. The percentage of cells in the G0/G1 phase, S phase, and G2/M phase were determined according to relative DNA content analyzed using a FACScan flow cytometer (Becton Dickinson, Franklin Lakes, NJ) [22].

### Immunodetection of KIAA0247

To further evaluate the highly expressed KIAA0247, the colonic cells transfected with shLuc or overKIAA0247 cultured in 6-well dishes were fixed, permeabilized, and blocked for immunofluorescent staining as previously reported, with some essential modifications [17]. Cells were probed with diluted anti-KIAA0247 antibody (1:500; H00009766-B01P; Abnova, Taipei, Taiwan) for 16 h at 4°C followed by incubation with R-phycoerythrin-conjugated goat anti-rabbit antibody (1:200; 405307; BioLegend, San Diego, CA) for 1 h at room temperature. The cellular DNA was stained with 4",6" diamidino-2-phenylindole. The stained samples were then dehydrated, mounted, and analyzed using a Nikon Eclipse 80i fluorescence microscope (Nikon Instruments, Melville, NY).

### Statistical analysis

Survival probabilities were estimated using the Kaplan-Meier method and compared using the log-rank test. Chi-squared or Fisher's exact tests were used for group comparisons. Kendall's tau-b correlation and linear regression analysis were applied to analyze correlations between the relative levels of fecal KIAA0247 and sizes of colonic tumor [23]. The Student's *t *test was used to compare the mRNA levels of cyclins in different groups. These statistical analyses were performed using SPSS 13.0 software (SPSS, Chicago, IL). The Medcalc software statistical package was employed to generate receiver-operating characteristic (ROC) curves. A *p *value < 0.05 was considered statistically significant.

## Results

### Expression of KIAA0247 in multiple gastrointestinal tissues and colonic cell lines

**Figure 1:**
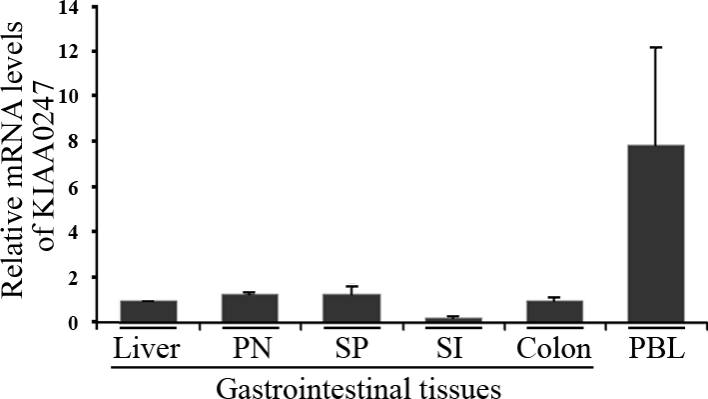
**Relative KIAA0247 mRNA levels in gastrointestinal tissues**. KIAA0247 mRNA levels quantified and normalized by individual levels of 18S rRNA. The organs of gastrointestinal tissues include the liver, pancreas (PN), spleen (SP), small intestine (SI), and colon. PBL, peripheral blood lymphocyte. Each KIA0247 mRNA level is relative to that in the liver. Data are representative of three independent experimental repeats.

qRT-PCR determined the expression of the uncharacterized gene, KIAA0247, in human gastrointestinal tissues and colonic cell lines. Results indicated that KIAA0247 ubiquitously expresses in gastrointestinal tissues and in peripheral blood leukocytes (PBL), with highest expression in PBL and lowest expression in the small intestine (Figure 1).

### Relationship of fecal KIAA0247 expression with clinical features of CRC patients

**Figure 2:**
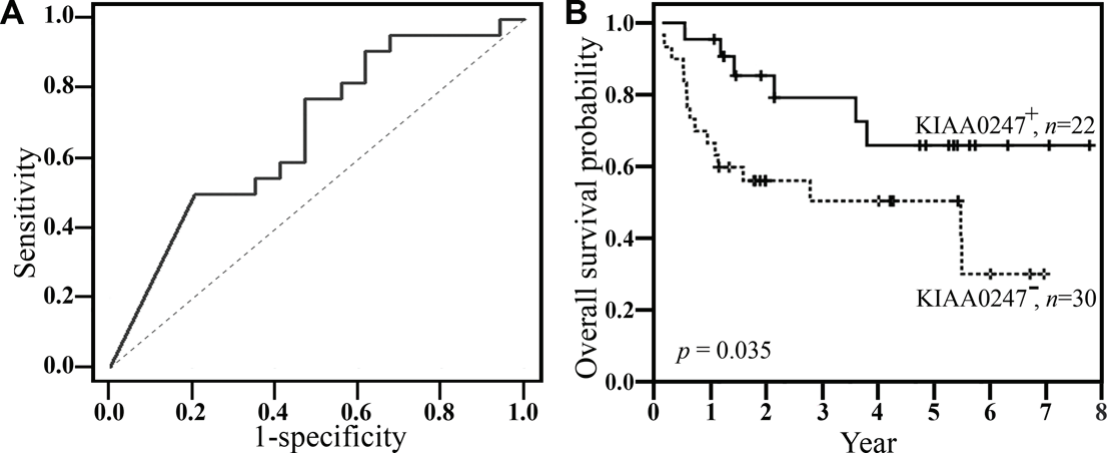
**Overall survival of CRC patients according to fecal KIAA0247 mRNA levels**. (A) Receiver operating characteristic curve for fecal KIAA0247 from CRC patients. (B) Overall survival of CRC patients. Survival probabilities estimated by the Kaplan-Meier method and compared using the log-rank test according to the fecal KIAA0247 mRNA levels in CRC patients. Patients are stratified into two groups: KIAA0247^- ^(<0.4897, *n *= 30) and KIAA0247^+ ^(≥0.4897, *n *= 22). *p *= 0.035, log-rank test.

**Figure 3:**
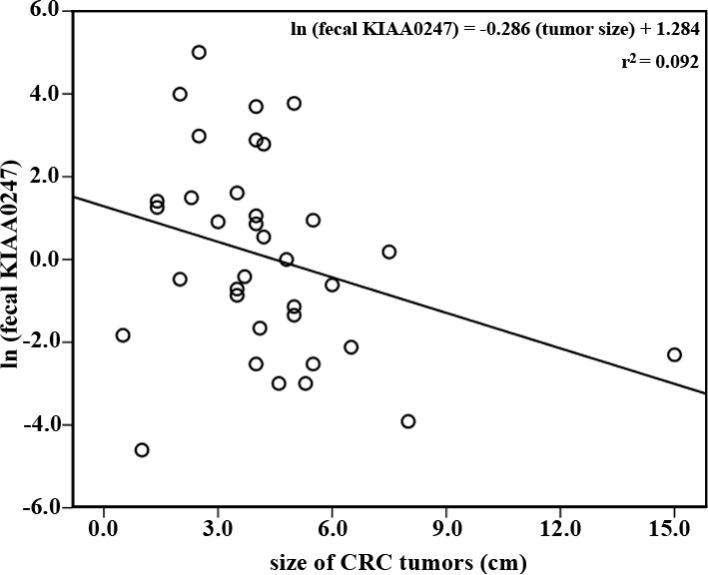
**Correlation between KIAA0247 fecal expression and sizes of CRC tumors**. The sizes of CRC tumors negatively associated with the natural logarithm of fecal KIAA0247 expression (slope = -0.286, *p *= 0.076).

Receiver-operating characteristic (ROC) curve analysis, based on relative KIAA0247 expression levels, stratified the 56 CRC patients into two groups to determine the clinical significance of fecal KIAA0247 expression. A cutoff at a fecal KIAA0247 expression level of 0.4897 provided a sensitivity of 0.77 (95% CI, 0.55-0.92) and a specificity of 0.53 (95% CI, 0.35-0.70) for predicting the prognosis of patients (*p *= 0.017). The area under the ROC curve for fecal KIAA0247 was 0.673 (95% CI, 0.535-0.793) (Figure 2A). The group with higher fecal KIAA0247 expression (KIAA0247^+^, *n *= 22; ≥ 0.4897) demonstrated a greater five-year overall survival rate than the group with lower fecal KIAA0247 expression (KIAA0247^-^, *n *= 30; < 0.4897) (66.0 ± 11.6%; *p *= 0.035, log-rank test) (Figure 2B). The Kendall's tau-b correlation test revealed an inverse relationship between fecal levels of KIAA0247 and the size of CRC tumors (Kendall's tau-b = -0.202; *p *= 0.047). Figure 3 shows this negative association, plotted according to linear regression (slope = -0.286), with almost statistical significance (*p *= 0.076). Table 3 also shows the association between fecal KIAA0247 and tumor size. A significantly higher percentage (56.7%, 17 of 30) of patients with positive fecal KIAA0247 occurred in the group in which patients had a tumor size smaller than the mean value (4.4 cm) (*p *= 0.020). Although no significant differences were noted for other clinical features (*p *> 0.05), the patients with positive fecal KIAA0247 demonstrated a trend to be diagnosed at an earlier stage (AJCC Stage I; 56.5%, 13 of 23; *p *= 0.061) and to have lower levels of serum carcinoembryonic antigen (≤ 5 ng/mL; 53.6%, 15 of 28; *p *= 0.072).

**Table 3:** The association between fecal KIAA0247 and clinical features

**Features**^a^	***n***^**b**^	Kendall's tau-b	*p*-value
Age (years)	55	0.020	0.836
			
CEA (ng/ml)	55	-0.076	0.430
			
CA19-9 (U/ml)	53	-0.076	0.440
			
Tumor size (cm)	51	-0.202	0.047

^a ^CEA, carcinoembryonic antign; CA19-9, carbohydrate antigen 19-9.

^b ^Numbers of assessed cases are dependent on the available cases.

### Reduction in proportion of colonic cells in G2/M phase with increased KIAA0247 expression

**Figure 4:**
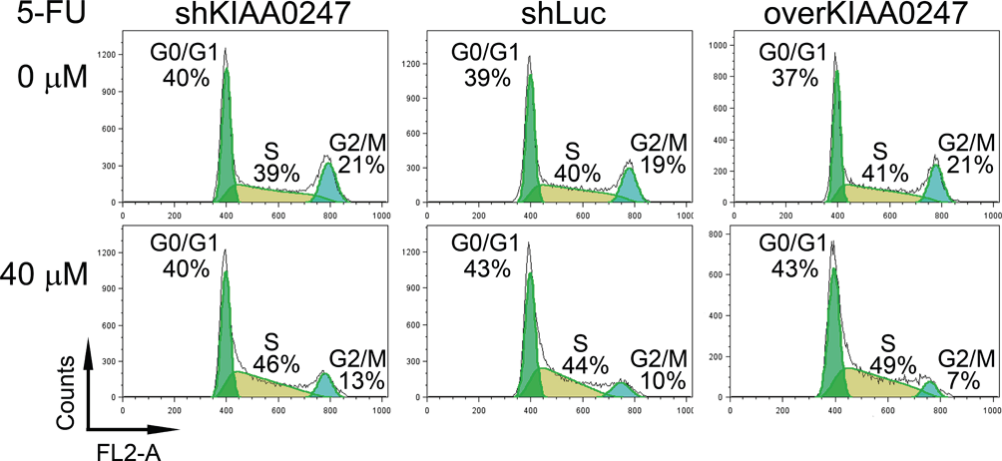
**Reduced proportions of colonic cells in G2/M phase according to KIAA0247expression and 5-FU treatment**. The p53-null HCT116 cells (HCT116 p53^-/-^) with varying KIA0247 expression stained with propidium iodide for evaluation of nuclei fluorescence. The percentages of cell numbers in the cell cycle phases are also shown. shKIAA0247, KIAA0247-silent cells; shLuc, control cells without changing the expression of KIAA0247; overKIAA0247, KIAA0247-overexpressing cells. 5-FU, 5-fluorouracil.

To exclude the influence of p53 on the cell cycle, a p53 knockdown CRC cell line (HCT116 p53^-/-^) revealed the cellular effects of KIAA0247 in the presence of 5-FU. DNA content staining determined the proportions of these colonic cells in G0/G1, S, and G2/M phases of the cell cycle. In these HCT116 p53^-/- ^cells, the proportion of cells in the G2/M phase was larger (13%) in KIAA0247-silent cells than in the respective shLuc control (10%) and KIAA0247-overexpressing cells (7%) after the addition of a low dose (40 μM) of 5-FU (Figure 4). The KIAA0247-overexpressing cells showed only one-third (7% *vs*. 21%) as many cells in the G2/M fraction after treatment with 40 μM 5-FU.

**Figure 5:**
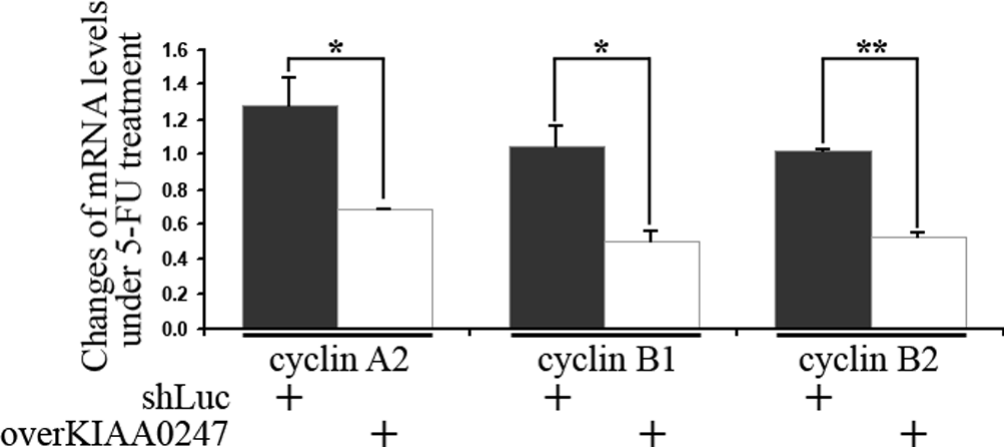
**Cyclin gene expression changes according to KIAA0247 expression and 5-FU treatment**. Individual levels of 18S rRNA in the p53-null HCT116 cells (HCT116 p53^-/-^) quantified and normalized cyclin mRNA levels. Relative expressions of cyclin genes (as indicated) acquired by comparing normalized mRNA levels of cyclins with 40 μM 5-FU treatment to those in 5-FU-free conditions. shLuc, control cells without changing the expression of KIAA0247; overKIAA0247, KIAA0247-overexpressing cells. 5-FU, 5-fluorouracil. The asterisks indicate *p < 0.05 and **p < 0.01. Data are representative of three independent experimental repeats.

To obtain a more comprehensive understanding of the ability of KIAA0247 to reduce the G2/M population, qRT-PCR quantified the mRNA levels of genes belonging to the highly conserved cyclin family. As shown in Figure 5, CRC cells that overexpressed KIAA0247 simultaneously downregulated the expression of three cyclin genes (cyclin A2, cyclin B1, and cyclin B2) after 40 μM 5-FU treatment. For example, the mRNA level of cyclin A2 in 5-FU-treated KIAA0247-overexpressing cells was 69% of that in these cells without 5-FU treatment. However, this cyclin A2 downregulation was not detected in the shLuc cells. Cyclin B1 and cyclin B2 mRNA levels demonstrated similar trends after the same treatment.

### Intracellular localization of KIAA0247 in colonic cells

**Figure 6:**
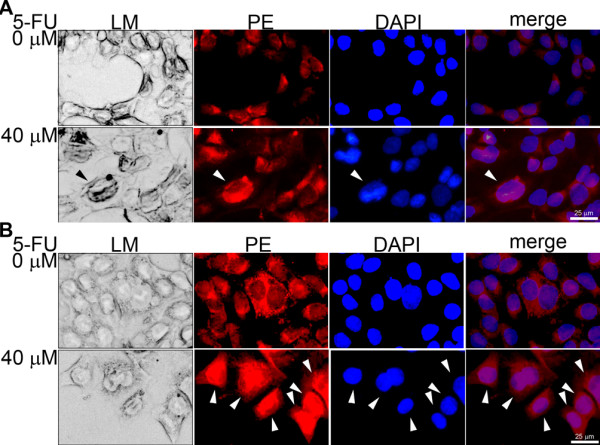
**Changes in intracellular localization according to KIAA0247 expression and 5-FU treatment**. Immunofluorescent staining in the p53-null HCT116 cells (HCT116 p53^-/-^). A) shLuc cells (200 ×) and B) overKIAA0247 cells (200 ×) stained for cellular KIAA0247 using diluted anti-KIAA0247 antibody. Secondary antibody, R-phycoerythrin (PE) -conjugated goat anti-rabbit antibody; DNA counterstaining, 4",6" diamidino-2-phenylindole (DAPI). LM, light microscope images; Merge, merged images from PE and DAPI. White arrowhead, cells with nuclear accumulation of KIAA0247; bars, 25 μm.

Immunofluorescent staining of overexpressed KIAA0247 in HCT116 p53^-/- ^cells identified that, under 5-FU-free conditions, the cytoplasm of CRC cells weakly expressed endogenous KIAA0247 (red fluorescence). This endogenous KIAA0247 demonstrated a tendency to move into the nucleus after treatment of cells with 40 μM 5-FU (Figure 6A, indicated as white arrowhead). In the KIAA0247-overexpressing cells KIAA0247 clearly accumulated in the nucleus (Figure 6B, indicated as white arrowhead). KIAA0247 overexpressed in the cytoplasm of most CRC cells without 5-FU treatment and accumulated in the nucleus after cellular DNA damage by 40 μM 5-FU.

## Discussion

Cell cycle checkpoints are important control mechanisms which ensure the proper passage of genetic codes and genome stability [24,25]. One of the checkpoints, the G2/M checkpoint, blocks the entry into mitosis after DNA damage [26]. Many previous reports indicated that p53 can regulate the G2/M transition via induction of p21 and 14-3-3σ [27,28] or associated apoptosis [29]. The findings of two investigations indicated that a p53-independent control also coordinates activation of the G2/M checkpoint [30,31].

This study demonstrated that KIAA0247 is under p53-independent control in CRC cells despite speculation that it is a p53-responsive target [12]. The predicted p53-responsive elements in the KIAA0247 promoter region demonstrated no electrophoretic mobility shift with p53 protein in a gel shift assay (data not shown). Higher expression of KIAA0247 occurred in fecal samples from early-stage CRC patients with a greater five-year overall survival rate. Use of a p53-null CRC cell line at Dukes' stage B, HCT116 p53^-/-^, as a target cell, excluded the influence of p53 on the cell cycle to correspond with the clinical findings.

Molecular markers are needed to assess CRC patients at Dukes' stage B who could benefit from adjuvant therapy [32]. Clinicians widely and routinely use 5-FU as one of the components in the therapeutic regimen [33,34] and a cytotoxic effect occurs during the intracellular metabolism of 5-FU. Such adjuvant chemotherapy is also beneficial to patients at Dukes' stage C [35]. In the present study's findings with a CRC cell line at Dukes' stage B, 40 μM 5-FU decreased the number of cells in G2/M in the presence of KIAA0247 expression. The presence of KIAA0247 expression and 5-FU also negatively modulated three common cell cycle activators. These data emphasize that early-stage CRC cells that are able to overexpress KIAA0247 could impede the progression of the cell cycle at the G2/M phase if an appropriate amount of 5-FU damages the cellular DNA.

The DNA damage response activates in precancerous lesions to permit CRC progression [36]. As reviewed by Wei et al., the prevention of DNA instability and uracil misincorporation might reduce the risk of the early transformative stages of CRC carcinogenesis [37]. Therefore, early during CRC carcinogenesis, an effective cytotoxic effect induced by 5-FU in the KIAA0247-expressing cells could be crucial in controlling the G2/M checkpoint and in decreasing the number of cells in G2/M. At the same time, reduced levels of cyclins would negatively control the cell cycle checkpoints. The combination of cell cycle arrest and downregulation of cyclins might suggest that patients with higher fecal KIAA0247 have smaller tumors because of a slowing of the progression of the cell cycle. Meanwhile, fecal KIAA0247 provides a suitable therapeutic indicator for CRC patients at Dukes' stage B in need of adjuvant 5-FU therapy. This study's data are partly consistent with another group's report that enhancing the cytotoxic effect of chemotherapeutic reagents inactivates the G2/M checkpoint leading to tumor cell death [24].

When testing the cDNA from multiple tissues, KIAA0247 expression was highest in PBL and at various levels in gastrointestinal tissues. These results suggest that fecal KIAA0247 provides a more useful therapeutic reference for early-stage CRC patients than blood KIAA0247. This translocation of KIAA0247 from the cytoplasm to the nucleus might be involved in the control of the G2/M checkpoint. The cellular effect of KIAA0247 is very similar to that of 14-3-3σ, whose overexpression could also cause G2/M cell cycle arrest, although 14-3-3σ is a p53-dependent inhibitor of G2/M progression [26].

In the group's previous studies of fecal gene expression, advanced microarray technology defined global changes in gene expression detectable in feces [18,38]. Results identified a novel gene for a homologue of the Drosophila headcase protein (HECA) as a classifier of early-stage CRC [38]. Comprehensive results for HECA and KIAA0247 indicate both fecal molecules could be markers of early-stage CRC. In this study, levels of fecal KIAA0247 inversely related to CRC tumor size with patients with high levels of fecal KIAA0247 having a longer five-year overall survival. Cell line results identifying that overexpressed KIAA0247 could move into the nucleus and repress the progression of the cell cycle at the G2/M phase supported the clinical findings. The downregulation of three cyclins may partly cause this repression. However, the exact mechanism by which KIAA0247 operates remains unclear. A high priority is to study other factors that lead to growth arrest, senescence, and apoptosis.

## Conclusions

This study describes and characterizes, for the first time, KIAA0247 from CRC patients using flow cytometry and qRT-PCR analysis. Results indicate that fecal KIAA0247 expression is a useful indicator of the need for 5-FU treatment in CRC, especially in cases diagnosed at early stages.

## List of abbreviations

CRC: colorectal cancer; 5-FU: 5-fluorouracil; qRT-PCR: quantitative real-time polymerase chain reaction; ROC: receiver-operating characteristic.

## Competing interests

The authors declare that they have no competing interests.

## Authors' contributions

CC Chang, SHY and CJH participated in the design of the study and carried out the molecular analyses. YCC and HHW performed the qRT-PCR, statistical analyses, and RNAi and overexpression of target gene, flow cytometry and immuno-analyses. CML and CLL participated in discussion, and CC Chien helped in the analyses of the experiments. SHY, SMH and CJH worked on the manuscript, and SHY and CJH also provided grant support for this study. All authors read and approved the final version of this manuscript.

## References

[B1] Lieberman D Progress and challenges in colorectal cancer screening and surveillance Gastroenterology 2010 138 2115 2126 10.1053/j.gastro.2010.02.006 20167216

[B2] Kim MS Lee J Sidransky D DNA methylation markers in colorectal cancer Cancer Metastasis Rev 2010 29 181 206 10.1007/s10555-010-9207-6 20135198

[B3] Allegra C Sargent D Molecular diagnostics: assays, tissues, progress, and pitfalls J Clin Oncol 2003 21 395 396 10.1200/JCO.2003.11.073 12560423

[B4] Lagerholm S Lagerholm S Dutta S Nair P Non-invasive detection of c-myc p64, c-myc p67 and c-erbb-2 in colorectal cancer Scand J Gastroenterol 2005 40 1343 1350 10.1080/00365520510023549 16334444

[B5] Vogelstein B Fearon ER Hamilton SR Genetic alterations during colorectal-tumor development N Engl J Med 1988 319 525 532 10.1056/NEJM198809013190901 2841597

[B6] Macarulla T Ramos FJ Capdevila J Saura C Tabernero J Novel targets for anticancer treatment development in colorectal cancer Clin Colorectal Cancer 2006 6 265 272 10.3816/CCC.2006.n.045 17241511

[B7] Voutsadakis IA Pathogenesis of colorectal carcinoma and therapeutic implications: the roles of the ubiquitin-proteasome system and Cox-2 J Cell Mol Med 2007 11 252 285 10.1111/j.1582-4934.2007.00032.x 17488476 PMC3822826

[B8] DiPaola RS To arrest or not to G(2)-M Cell-cycle arrest: commentary re: A. K. Tyagi et al., Silibinin strongly synergizes human prostate carcinoma DU145 cells to doxorubicin-induced growth inhibition, G(2)-M arrest, and apoptosis. Clin. cancer res., 8: 3512-3519, 2002 Clin Cancer Res 2002 8 3311 3314 12429616

[B9] Owa T Yoshino H Yoshimatsu K Nagasu T Cell cycle regulation in the G1 phase: a promising target for the development of new chemotherapeutic anticancer agents Curr Med Chem 2001 8 1487 1503 11562278 10.2174/0929867013371996

[B10] Carlson B Lahusen T Singh S Loaiza-Perez A Worland PJ Pestell R Albanese C Sausville EA Senderowicz AM Down-regulation of cyclin D1 by transcriptional repression in MCF-7 human breast carcinoma cells induced by flavopiridol Cancer Res 1999 59 4634 4641 10493518

[B11] Hirose Y Berger MS Pieper RO Abrogation of the Chk1-mediated G(2) checkpoint pathway potentiates temozolomide-induced toxicity in a p53-independent manner in human glioblastoma cells Cancer Res 2001 61 5843 5849 11479224

[B12] Robles AI Bemmels NA Foraker AB Harris CC APAF-1 is a transcriptional target of p53 in DNA damage-induced apoptosis Cancer Res 2001 61 6660 6664 11559530

[B13] Staib F Robles AI Varticovski L Wang XW Zeeberg BR Sirotin M Zhurkin VB Hofseth LJ Hussain SP Weinstein JN The p53 tumor suppressor network is a key responder to microenvironmental components of chronic inflammatory stress Cancer Res 2005 65 10255 10264 10.1158/0008-5472.CAN-05-1714 16288013 PMC1421332

[B14] Kanaoka S Yoshida K Miura N Sugimura H Kajimura M Potential usefulness of detecting cyclooxygenase 2 messenger RNA in feces for colorectal cancer screening Gastroenterology 2004 127 422 427 10.1053/j.gastro.2004.05.022 15300574

[B15] Kawada M Mizuno M Nasu J Uesu T Okazaki H Okada H Shimomura H Yamamoto K Tsuji T Fujita T Release of decay-accelerating factor into stools of patients with colorectal cancer by means of cleavage at the site of glycosylphosphatidylinositol anchor J Lab Clin Med 2003 142 306 312 10.1016/S0022-2143(03)00137-9 14647034

[B16] Yang SH Chien CC Chen CW Li SY Huang CJ Potential of faecal RNA in diagnosing colorectal cancer Cancer Lett 2005 226 55 63 10.1016/j.canlet.2004.11.005 16004932

[B17] Chang CC Yang SH Chien CC Chen SH Pan S Lee CL Lin CM Sun HL Huang CC Wu YY Clinical meaning of age-related expression of fecal cytokeratin 19 in colorectal malignancy BMC Cancer 2009 9 376 10.1186/1471-2407-9-376 19849844 PMC2776602

[B18] Huang CJ Chien CC Yang SH Chang CC Sun HL Cheng YC Liu CC Lin SC Lin CM Faecal ribosomal protein L19 is a genetic prognostic factor for survival in colorectal cancer J Cell Mol Med 2008 12 1936 1943 18266979 10.1111/j.1582-4934.2008.00253.x PMC4506161

[B19] Yang RN Yang SH Chang CC Chien CC Pan S Huang CJ Upregulation of fecal cytokeratin 19 is associated with prognosis in older colorectal cancer patients Genet Test Mol Biomarkers 2010 14 703 708 10.1089/gtmb.2010.0047 20854102

[B20] Li J Tan J Zhuang L Banerjee B Yang X Chau JF Lee PL Hande MP Li B Yu Q Ribosomal protein S27-like, a p53-inducible modulator of cell fate in response to genotoxic stress Cancer Res 2007 67 11317 11326 10.1158/0008-5472.CAN-07-1088 18056458

[B21] Miyake H Hanada N Nakamura H Kagawa S Fujiwara T Hara I Eto H Gohji K Arakawa S Kamidono S Overexpression of Bcl-2 in bladder cancer cells inhibits apoptosis induced by cisplatin and adenoviral-mediated p53 gene transfer Oncogene 1998 16 933 943 10.1038/sj.onc.1201602 9484785

[B22] Cubas R Zhang S Li M Chen C Yao Q Trop2 expression contributes to tumor pathogenesis by activating the ERK MAPK pathway Mol Cancer 2010 9 253 10.1186/1476-4598-9-253 20858281 PMC2946292

[B23] Chang CC Chien CC Yang SH Chen SH Huang CJ Identification and Clinical Correlation of Decreased Expression of Cytoplasmic Dynein Heavy Chain 1 in Patients with Colorectal Cancer Clin Mol Medicine 2008 1 6 10

[B24] Fingerle-Rowson G Petrenko O MIF coordinates the cell cycle with DNA damage checkpoints. Lessons from knockout mouse models Cell Div 2007 2 22 10.1186/1747-1028-2-22 17640378 PMC1941730

[B25] Yeh YH Huang YF Lin TY Shieh SY The cell cycle checkpoint kinase CHK2 mediates DNA damage-induced stabilization of TTK/hMps1 Oncogene 2009 28 1366 1378 10.1038/onc.2008.477 19151762

[B26] Taylor WR Stark GR Regulation of the G2/M transition by p53 Oncogene 2001 20 1803 1815 10.1038/sj.onc.1204252 11313928

[B27] Agarwal ML Agarwal A Taylor WR Stark GR p53 controls both the G2/M and the G1 cell cycle checkpoints and mediates reversible growth arrest in human fibroblasts Proc Natl Acad Sci USA 1995 92 8493 8497 10.1073/pnas.92.18.8493 7667317 PMC41183

[B28] Hermeking H Lengauer C Polyak K He TC Zhang L Thiagalingam S Kinzler KW Vogelstein B 14-3-3 sigma is a p53-regulated inhibitor of G2/M progression Mol Cell 1997 1 3 11 10.1016/S1097-2765(00)80002-7 9659898

[B29] Concin N Stimpfl M Zeillinger C Wolff U Hefler L Sedlak J Leodolter S Zeillinger R Role of p53 in G2/M cell cycle arrest and apoptosis in response to gamma-irradiation in ovarian carcinoma cell lines Int J Oncol 2003 22 51 57 12469184

[B30] Bache M Dunst J Wurl P Frode D Meye A Schmidt H Rath FW Taubert H G2/M checkpoint is p53-dependent and independent after irradiation in five human sarcoma cell lines Anticancer Res 1999 19 1827 1832 10470122

[B31] Chung JH Bunz F Cdk2 is required for p53-independent G2/M checkpoint control PLoS Genet 2010 6 e1000863 10.1371/journal.pgen.1000863 20195506 PMC2829054

[B32] Gangadhar T Schilsky RL Molecular markers to individualize adjuvant therapy for colon cancer Nat Rev Clin Oncol 2010 7 318 325 10.1038/nrclinonc.2010.62 20440283

[B33] Wang BD Kline CL Pastor DM Olson TL Frank B Luu T Sharma AK Robertson G Weirauch MT Patierno SR Prostate apoptosis response protein 4 sensitizes human colon cancer cells to chemotherapeutic 5-FU through mediation of an NF kappaB and microRNA network Mol Cancer 2010 9 98 10.1186/1476-4598-9-98 20433755 PMC2883962

[B34] Sasaki K Tsuno NH Sunami E Tsurita G Kawai K Okaji Y Nishikawa T Shuno Y Hongo K Hiyoshi M Chloroquine potentiates the anti-cancer effect of 5-fluorouracil on colon cancer cells BMC Cancer 2010 10 370 10.1186/1471-2407-10-370 20630104 PMC2914703

[B35] Cunningham D Atkin W Lenz HJ Lynch HT Minsky B Nordlinger B Starling N Colorectal cancer Lancet 2010 375 1030 1047 10.1016/S0140-6736(10)60353-4 20304247

[B36] Oka K Tanaka T Enoki T Yoshimura K Ohshima M Kubo M Murakami T Gondou T Minami Y Takemoto Y DNA damage signaling is activated during cancer progression in human colorectal carcinoma Cancer Biol Ther 2010 9 246 252 10.1158/1535-7163.MCT-09-0495 20023412 PMC2977911

[B37] Wei EK Wolin KY Colditz GA Time course of risk factors in cancer etiology and progression J Clin Oncol 2010 28 4052 4057 10.1200/JCO.2009.26.9324 20644083 PMC4872328

[B38] Chien CC Chang CC Yang SH Chen SH Huang CJ A homologue of the Drosophila headcase protein, HECA, is a novel tumor marker for early-stage colorectal cancer Oncol Rep 2006 15 919 926 16525680

